# Development and characterization of chimera of yellow fever virus vaccine strain and Tick-Borne encephalitis virus

**DOI:** 10.1371/journal.pone.0284823

**Published:** 2023-05-10

**Authors:** Nadezhda Kuznetsova, Andrei Siniavin, Alexander Butenko, Victor Larichev, Alina Kozlova, Evgeny Usachev, Maria Nikiforova, Olga Usacheva, Alexey Shchetinin, Andrei Pochtovyi, Elena Shidlovskaya, Alina Odintsova, Elizaveta Belyaeva, Aleksander Voskoboinikov, Arina Bessonova, Lyudmila Vasilchenko, Galina Karganova, Vladimir Zlobin, Denis Logunov, Vladimir Gushchin, Alexander Gintsburg

**Affiliations:** 1 Federal State Budgetary Institution "National Research Centre for Epidemiology and Microbiology named after the Honorary Academician N. F. Gamaleya" of the Ministry of Health of the Russian Federation, Moscow, Russia; 2 Department of Molecular Neuroimmune Signalling, Shemyakin-Ovchinnikov Institute of Bioorganic Chemistry, Russian Academy of Sciences, Moscow, Russia; 3 Department of Virology, Lomonosov Moscow State University, Moscow, Russia; 4 Laboratory of Biology of Arboviruses, FSASI Chumakov Federal Scientific Center for Research and Development of Immune-and-Biological Products of RAS, Moscow, Russia; 5 Institute for Translational Medicine and Biotechnology, Sechenov University, Moscow, Russia; 6 Department of Infectiology and Virology, Federal State Autonomous Educational Institution of Higher Education I M Sechenov First Moscow State Medical University of the Ministry of Health of the Russian Federation (Sechenov University), Moscow, Russia; University of Pécs: Pecsi Tudomanyegyetem, HUNGARY

## Abstract

Tick-borne encephalitis virus (TBEV) is one of the most threatening pathogens which affects the human central nervous system (CNS). TBEV circulates widely in Northern Eurasia. According to ECDC, the number of TBE cases increase annually. There is no specific treatment for the TBEV infection, thus vaccination is the main preventive measure. Despite the existence of several inactivated vaccines currently being licensed, the development of new TBEV vaccines remains a leading priority in countries endemic to this pathogen. Here we report new recombinant virus made by infectious subgenomic amplicon (ISA) approach using TBEV and yellow fever virus vaccine strain (YF17DD-UN) as a genetic backbone. The recombinant virus is capable of effective replication in mammalian cells and induce TBEV-neutralizing antibodies in mice. Unlike the original vector based on the yellow fever vaccine strain, chimeric virus became neuroinvasive in doses of 10^7^−10^6^ PFU and can be used as a model of flavivirus neuroinvasiveness, neurotropism and neurovirulence. These properties of hybrid structures are the main factors limiting their practical use as vaccines platforms.

## Introduction

Tick-borne encephalitis (TBE) is one of the most severe arbovirus diseases which affects the human central nervous system (CNS). This virus is quite widespread in natural foci in Europe and Northeast Asia [1]. There is an annual increase in the number of cases of infection, according to ECDC data [2]. In Austria, Germany, Switzerland, Lithuania and the Czech Republic, the increase was higher compared to the average for the previous three years (P<0.05) with an increase of 88%, 48%, 51%, 28% and 18%, respectively. Six countries reported ≥5 cases of tick-borne encephalitis per 100,000 people, which is specified as an endemic disease by the World Health Organization (WHO). Possible factors contributing to this surge may include more active participation in outdoor activities in endemic regions and an increase in the number of ticks and their activity. There is no specific treatment against Tick-borne encephalitis virus (TBEV). Therefore, WHO recommends vaccination against TBE in all age groups, including children, in endemic regions [3].

Generating chimeric vaccines is of great interest. The genome of the live attenuated 17D vaccine has been suggested as a genetic backbone in developing vaccines against other viruses. Many of such viruses are flaviviruses, including West Nile virus (WNV), Japanese encephalitis virus (JEV), TBEV, Zika virus and Dengue virus. Since 2012, two vaccines based on 17D-204 (ChimeriVax technology) have been approved for use. Imojev™ and Dengvaxia® to prevent Japanese encephalitis (JE) and Dengue, respectively [4–9]. Candidate vaccines for West Nile virus and Zika virus are also being developed using ChimeriVax technology [10–14]. Because of its genetic stability, the genome of the 17D vaccine strain has also been used to develop candidate vaccines against unrelated pathogens such as Plasmodium falciparum, Trypanosoma cruzi, HIV, Lassa virus and SARS-CoV-2 [15–19]. Many of these candidates and vaccines work well, as they stimulate both humoral and cellular immune response. Antibodies obtained as a result of vaccination are virus-neutralizing, and infectious animal models show the protectiveness of candidate vaccines [15–20].

The approach to construct candidate vaccines based on a flavivirus template, including YFV, is a simple and universal method of reverse genetics “ISA” (infectious subgenomic amplicons). This method allows the generation of RNA viruses from genomic cDNA without cloning, cDNA reproduction in bacteria, or in vitro RNA transcription [21–24]. In our work, we designed a live chimeric YFV 17DD-UN/TBEV using a vaccine strain of the yellow fever virus 17DD as a genetic backbone.

## Materials and methods

### Strains of microorganisms, cell cultures and plasmid vectors used

Developing the chimeric constructs based on the YFV 17DD with prM and E TBEV genes inserts, we used TBEV strain 493 of the European subtype (Access ID GenBank OQ435379). The genome of the attenuated strain YFV 17DD (Access ID GenBank DQ100292.1) was obtained using de novo gene synthesis (Evrogen Ltd, Russia). For mouse challenge was used TBEV strain Absettarov of the European subtype (Access ID GenBank KU885457).

Cell cultures used in work: Vero E6 (ATCC CRL-1586), BHK-21 (ATCC CCL-10), MA-104 (ATCC CRL-2378.1), HEK293 (ATCC CRL-1573), Spev (BioLot, Russia) and LLC-MK2 (ATCC CCL-7). Cells were cultivated at 37°C and 5% СО_2_ in a complete DMEM growth medium (PanEco, Russia) supplemented with 5% FBS (Fetal bovine serum; HyClone, USA) and 1% penicillin-streptomycin solution (Gibco, USA).

For the development of various genetic constructs, the following plasmid vectors were used: in-house vector pBADmini (Ori+CmR) derived from pBAD33 (CmR) was used to clone parts of the YFV genome and the pEGFP-C1 vector was used to clone regulatory regions that ensure virus replication (CMV promoter, 5’ UTR, 3’UTR, HDR ribozyme, SV40 polyA signal). We carried all molecular cloning out using the E. coli strain Top10.

### Construction of infectious cDNA clones

Complete YFV genome, flanked with human cytomegalovirus promoter (pCMV) at its 5’-end and with hepatitis delta ribozyme, followed by the monkey virus 40 polyadenylation signal (HDR/SV40pA), at its 3’-end, was synthesized de novo (Evrogen, Russia) and amplified using a PCR method into three overlapping DNA fragments containing 5800, 3688 and 5784 base pairs.

Amplicons were received with a 2× Platinum SuperFi Green MasterMix kit (Thermo Fisher Scientific, USA) following the manufacturer’s instructions. We conducted plasmid construction and assembly with a Gibson Assembly Ultra MasterMix kit (Codex DNA, USA) following the manufacturer’s instructions. Selection of oligonucleotide primers for constructing cDNA infectious clones was carried out using the SnapGene program (Version 5.0).

Identification of the viable virus that is capable of replication was carried out after assembly using qRT-PCR. We selected oligonucleotide primers and probes for the non-structural protein NS5 gene of the backbone-YFV and Envelope protein (E gene) TBEV inserts, the oligonucleotides structure was as follows: YFV forward primer NS5F 5’-GCG GTA TCT TGA GTT TGA GG-3’, reverse primer NS5R 5’-AGG TCT CTG ATC ACA TAT CCT AG-3’, probe NS5TM 5’-FAM-AGC CAA TGC CTTC CAC TCC TCC TC-BHQ1-3’ and TBEV forward primer TBEV-E-F 5’-ACA CAC GGG AGA CTA TGT TG-3’, reverse primer TBEV-E-R 5’-TCT GAA GAA ACT GTG AAG GAT G-3’, probe TBEV-Z-eYF 5’-R6G-CGC AAA CGA GAC ACA TAG TGG GAG G-BHQ1-3’.

To test the virus-containing liquid, qPCRmix-HS and One Tube RT-PCR (Evrogen Ltd., Russia) were used following the instructions. The total volume of the reaction mixture was 25 μl. Amplification was performed using the QuantStudio 5 device (Applied Biosystems, USA). The following amplification program was specified: 50°C—30 min, 95°C—5 min (warm–up); 95°C—10 s / 60°C—20 s (detection) / 72°C—30 s—35 cycles. We carried out the selection of oligonucleotides using the SnapGene program (Version 5.0).

### High-throughput sequencing of genetic constructs and genomes of recombinant viruses

DNA libraries were created from plasmid DNA using the NEBNext Fast DNA Fragmentation & Library Prep Set for Ion Torrent (New England Biolabs, USA) following the manufacturer’s instructions. Libraries were sequenced on the Ion S 5XL platform (Thermo Fisher Scientific, USA). The raw data was filtered by quality and length using search v 2.17.0 and was collected de novo using SPAdes v3.14.0 with the—iontorrent key.

Viral RNA was fragmented, and the first cDNA chain was synthesized with random hexamer primers using the RevertAid First Strand cDNA Synthesis Kit (Thermo Fisher Scientific, USA), followed by the second DNA chain synthesis using NEBNext Ultra II Non-Directional RNA Second Strand Synthesis Module (New England Biolabs, USA). The obtained cDNA was used to prepare a DNA library with reagents supplied in the NEBNext Fast DNA Library Prep Set for Ion Torrent (New England Biolabs, USA). We sequenced libraries on the Ion S 5XL platform (Thermo Fisher Scientific, USA). The raw data was filtered by quality and length using vsearch v2.17.0. It was followed by mapping to the reference genome of the yellow fever virus vaccine strain 17DD (Access ID GenBank DQ100292.1) using the algorithm BWA-MEM v0.7.17-r1188. We carried out filtering and creation of a consensus sequence using cf tools v1.12 and bedtools v2.30.0.

### Mammalian cell transfection and estimation of CPE (cytopathic effect) and virus replication kinetics

To assemble infectious viruses, including hybrid constructions, transfection was performed using HEK293 or Vero E6 cells. For this purpose, cells were seeded into 48-well plates (2×10^5^ cells/well) the day before the experiment. Then, cell transfection was performed in OptiMem (Gibco, USA) medium using Transporter 5 transfection reagent (PEI; Polyscience) or Lipofectamine 2000 (LF2000; ThermoFisher, USA).

To determine the virus titer, Vero E6 cells were seeded into 24-well plates (2.5×10^5^cells/well) the day before the experiment. Next, various dilutions of the virus were added to the cell monolayer. After virus adsorption, medium was removed, and cells were overlaid with 0.3% Noble Agar (Sigma, USA) solution in DMEM (1:1). Plates were incubated for 4–5 days at 37°C and 5% СО_2_. Thereafter, cells were fixed with 5% PFA solution and stained with 1% crystal violet solution. We characterized the titer of the virus in PFU/ml (plaque-forming units) [25].

To assess the replicative activity of viruses, cells (Vero E6, BHK-21, MA-104, Spev or LLC-MK2) were seeded into 6-well plates (10^6^ cells/well) and infected with the corresponding virus (YFV 17DD or YFV/TBEV) at MOI 0.01 (multiplicity of infection). The kinetics of virus replication was analyzed for 4 days using qPCR and PFU assay. The quantity of viral RNA was calculated by qPCR using plasmid DNA with known concentrations as an external standard.

### Enzyme immunoassay (ELISA) to detect virus antigens

Plate wells were sensibilized in a volume of 100 μl per well with polyclonal specific and control immunoglobulins diluted in 0.05 M carbonate-bicarbonate buffer (CBB) with a pH of 9.6. Plates were incubated for 2 hours at 37°C. The unabsorbed immunoglobulin was removed by washing the wells twice with 0.01 M phosphate buffer solution pH 7.4 and 0.05% tween-20 (FBR-t). 150 μl 1% bovine serum albumin on FBR-t (FBR-t-A) was added and wells were incubated for 45 min at 37°C to block the surface of the plate wells that were not covered with globulins. Then, the FBR-t-A was removed from the plate.

The test samples diluted in FBR-t-A were introduced in parallel to 2 wells (each sample was tested with normal and specific immunoglobulins) in a volume of 100 μl and incubated for 1 hour at a temperature of 37°C. Then the plates were washed three times by the FBR-T. Antiviral conjugate (mouse antiviral antibodies labeled with horseradish peroxidase) was introduced into all wells of the plate at 100 μl per well and incubated for 1 hour at 37°C. The plate was washed 6 times by the FBR-T. To identify the peroxidase label, 100 ml of indicator solution (1 mm tetramethylbenzidine solution, 0.01% hydrogen peroxide in citrate buffer (pH4.0) was introduced into the wells of the plate. After 10 minutes, we added 100 ml of 1N sulfuric acid to all the wells in the plate. We evaluated the reaction in the μ-Quant device (Bio-Tek Instruments, USA) at a wavelength of 450 nm with the reference wavelength of 620 nm. The result was positive if the optical density (OD) of the test sample with a specific immunoglobulin in a well was greater than 0.3, 3 or higher than the OD of the same sample with normal immunoglobulin in a well. At the same time, the OD of this sample with normal immunoglobulin in the well did not exceed 0.25. Sucrose-acetone antigens of the corresponding viruses served as positive controls.

### Animal models

All animal procedures were conducted in accordance with the relevant guidelines for the care and use of laboratory animals and approved by the Local Ethics Commission of the National Research Center for Epidemiology and Microbiology named after Honorary Academician N.F. Gamaleya (protocol # 2, 29 December 2020). Animals were purchased from Stolbovaya nursery for laboratory animals (Russia). All animals were housed in separate cages (10 mice in cage) with controlled temperature (20–24°C) and humidity (45–65%). Mice were fed with a balanced rodent diet and water ad libitum during the entire study.

To access the pathogenicity and residual neuroinvasiveness of generated viruses, BALB/c female mice with a weight of 18–20 grams had been subcutaneously injected with various doses (in PFU) of a YFV 17DD-UN and YFV 17DD-UN/TBEV (4 groups, n = 10). Control groups included mice treated with 100 μl of growth medium Opti-MEM (placebo group, n = 10), TBEV (strain Absettarov, TBEV group, n = 10) and EnceVir (inactivated vaccine based on the Far-Eastern TBEV strain, JSC NPO Mikrogen, Russia) (vaccine group, n = 10). Animals were vaccinated by YFV 17DD-UN and YFV 17DD-UN/TBEV 1 time. Mice in the EnceVir group were immunized with the 10-fold diluted in Opti-MEM medium vaccine, animals were vaccinated 3 times with an interval between injections of 7 days [26]. The progression of clinical symptoms in animals has been observed for 28 days. Mice health and behavior were monitored once a day during the first week after inoculation and then twice a day till the end of the study. In case of the observation of neurological signs such as paresis mice were sacrificed by cervical dislocation and brain and liver were excised for subsequent qRT-PCR analysis. After 28 days of observation, survived animals were anaesthetized by intramuscular Zoletil-xylazine (45 mg/kg and 7.5 mg/kg, correspondingly) injection. Blood samples were collected in clot activator tubes (VACUETTE®, Greiner Bio-One, Austria) using heart puncture. Blood serum was collected in order to determine the level of neutralizing antibodies in the virus-neutralization assay.

To analyze the protective properties of the recombinant YFV 17DD-UN/TBEV virus, we infected immunized mice with TBEV strain Absettarov. BALB/c female mice with a weight of 18–20 grams were immunized with two dose (10^6^ and 10^5^ PFU) of a YFV 17DD-UN/TBEV (2 groups, n = 20), control groups mice were immunized with 100 μl of growth medium Opti-MEM (placebo group, n = 20) and EnceVir (vaccine group, n = 20). Four weeks after immunization animals all groups were inoculated by TBEV with a dose 2 LD_50_ and 400 LD_50_ (each group, n = 10). The LD_50_ was calculated using the Reed and Mench method [27]. Mice health and behavior were monitored once a day during four weeks after immunization and the first week after TBEV inoculation and then twice a day till the end of the study. In case of the observation of neurological signs such as paresis mice were sacrificed by cervical dislocation. After 28 days of observation, survived animals were sacrificed by cervical dislocation.

### Evaluation of serum neutralizing activity after chimeric YFV/TBEV immunization

Different dilutions of serum from immunized animals were mixed in equal proportion with 100TCID50 of TBEV strain Absettarov of the European subtype (Access ID GenBank KU885457) and incubated for 1 hour at 37°C. After incubation, serum-virus mix was transferred into a 96-well plate to Spev cells. Plates were incubated at 37°C and 5% СО_2_ for 4 days. The last dilution of the serum that showed full protection against the virus-induced cytopathic effect (CPE) was taken as the viral neutralizing titer.

## Results

### Generation of an infectious YFV 17DD strain using de novo synthesis and ISA method

**Fig 1:**
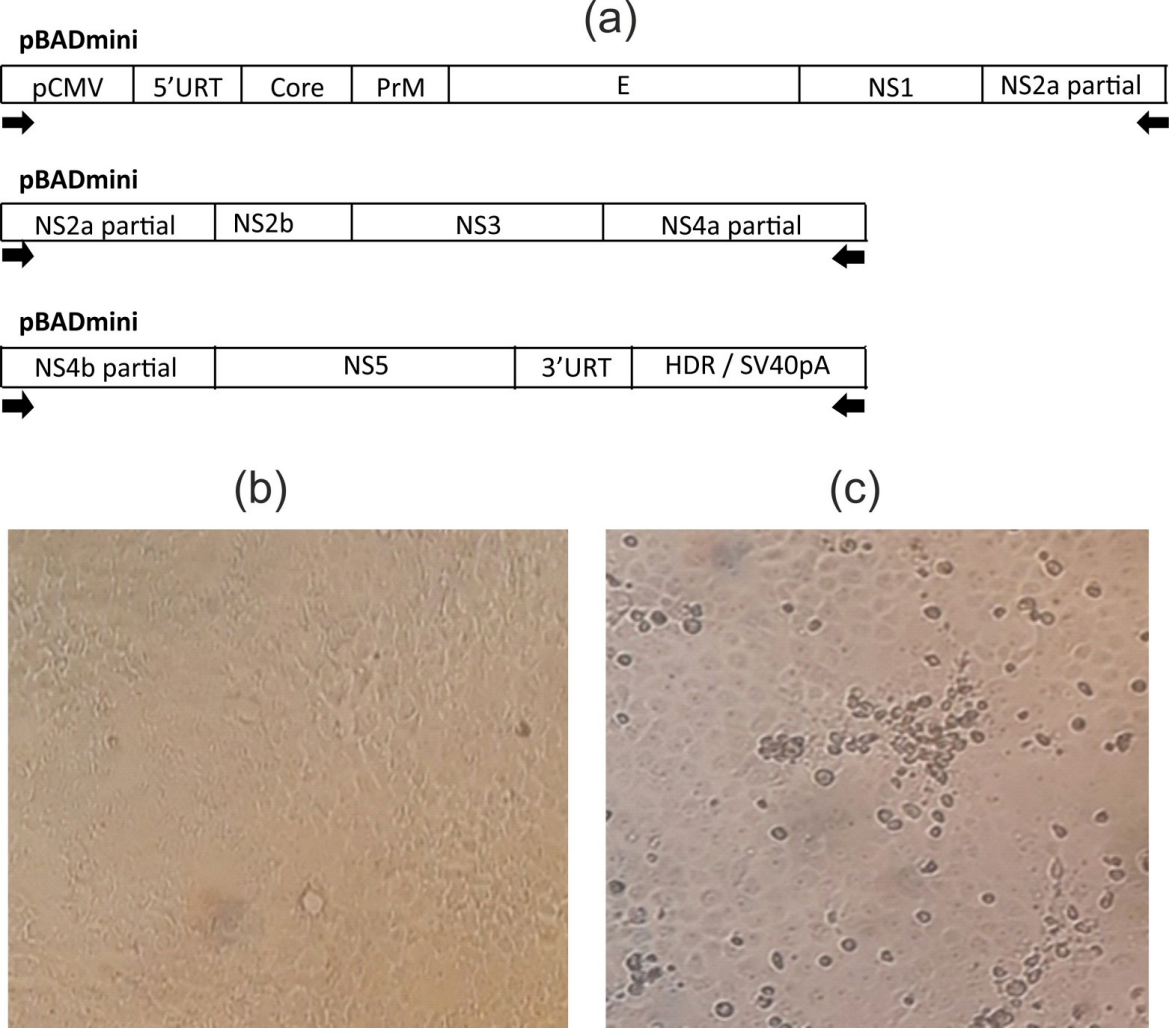
Construction of YFV 17DD-UN. (a) The layout of the regulatory region, structural and non-structural genes of YF 17DD in 3 pBADmini plasmids. The arrows show the sites of oligonucleotides for amplification of fragments of the YF genome before ISA full genome assembly protocol. (b) Control of VeroE6 cells without virus. (c) A virus-induced CPE of VeroE6 cells infected with the YF 17DD-UN.

To generate the YFV 17DD infectious clone that could be further used as a backbone for the ongoing development of chimeric flaviviruses, cDNA of the 17DD YFV strain was synthesized de novo. We identified that successful transfection and subsequent live virus generation could be achieved with three YFV 17DD amplicons. The first and third fragments are flanked at 5’ and 3’- ends with pCMV promoter and hepatitis delta ribozyme with HDR/SV40pA, respectively. Later three amplicons were cloned in a plasmid to generate stable constructs and to carry on the development of recombinant YFV/TBEV structure. We cloned all three fragments into the low copy number plasmid pBadmini. The retrieved structures were tested by Sanger and high-throughput DNA sequencing. The layout of the regulatory region, structural and non-structural genes of YFV 17DD in 3 pBADmini plasmids are represented in Fig 1A.

Using the classical ISA approach and plasmid-cloned parts of the YF virus by transfection of Vero E6 cells we generated the YF 17DD live virus. A virus-induced cytopathic effect (CPE) was observed in Vero E6 cells both after transfection and after virus passages (Fig 1B and 1C). To confirm virus replication and exclude the detection of amplicon or plasmid residues used for cell transfection, we performed real-time PCR in two versions, qPCRmix-HS (only hot start Taq polymerase) and One Tube RT-PCR (M-MLV reverse and Taq polymerase). The 10 cycles difference in Ct (cycle threshold) value between qPCRmix-HS (Ct 30) and One Tube RT-PCR (Ct 20) allows us to conclude the live infectious YF virus and its active replication in the cells. In the result of the ISA method, we assembled the yellow fever virus that was specified as YFV 17DD-UN.

After six passages in Vero E6 cells, the presence of the full YFV 17DD-UN genome has been confirmed by high-throughput sequencing. In accordance with a comparative analysis of the complete genomes of 17DD YFV (DQ100292.1) and YFV 17DD-UN the presence of three significant (at positions p.Ala2253Val, p.Ser2896Asn and p.His3222Arg) and four non-significant (at positions c.265T>C, c.370T>C, c.1051T>C and c.5305G>A) substitution was found (accession OP796361).

### Development of a chimeric YFV 17DD-UN/TBEV with the PreM/E TBEV insert

**Fig 2:**
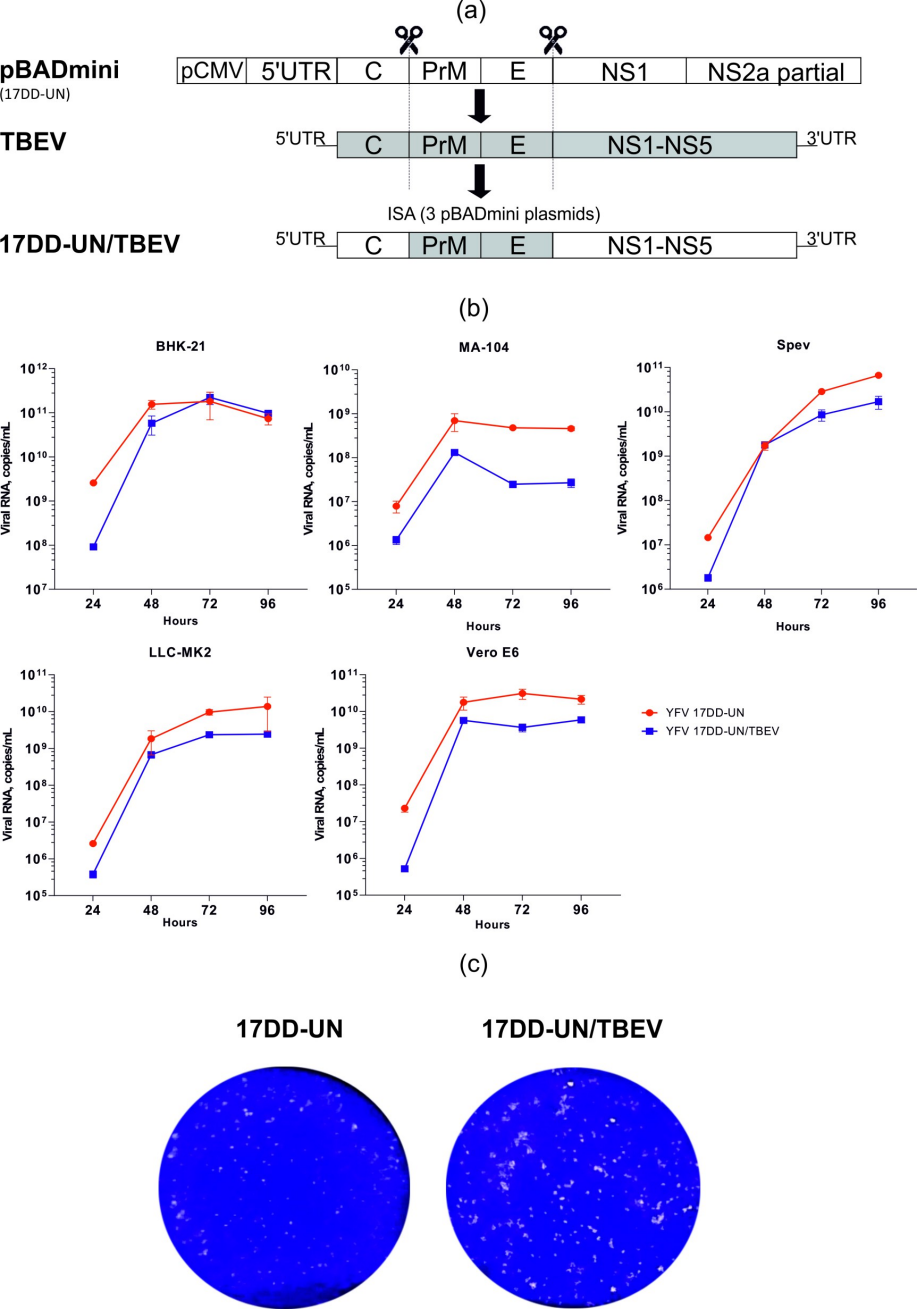
Construction of chimeric YFV 17DD-UN/TBEV. (a) Scheme for generation chimeric YFV 17DD-UN/TBEV. (b) Replication kinetics of parental and chimeric viruses. (c) Plaque assay of YFV 17DD-UN/TBEV, TBEV and YFV 17DD-UN.

YFV 17DD-UN strain served as a backbone in the development of the chimeric virus. The structural genes PrM and E of backbone YFV 17DD-UN were replaced by the same genes of TBEV. One of three constructs (pBadmini plasmids) containing Core, PrM, E, NS1 and part NS2a of YFV 17DD-UN was used in work (Fig 2A). We designed primers after selecting the junction sites of the viruses, which allowed the assembly of the chimeric virus using the Gibson method (Gibson assembly) with subsequent transformation into bacterial cells. Sanger sequencing confirmed the plasmid assembly and intergenic junctions of the two viruses.

The following transfection and passages in Vero E6 cells showed that the construction of the chimeric virus YFV 17DD-UN/TBEV was successful. We observed CPE on the cell line. Additionally, replication of virus was confirmed by qRT-PCR. The third and six passages of the hybrid virus were used for stability assessment by high-throughput sequencing. In the result of a high-throughput sequencing, a clone of the YFV 17DD-UN/TBEV hybrid with minimal significant replacements was selected. The virus-containing liquid collected from Vero E6 cells was also examined using enzyme immunoassay (ELISA), which confirmed the production of PreM and E TBEV antigen proteins by a chimeric virus (Table 1). To conduct the ELISA, plate wells were sensibilized with immunoglobulins against TBEV (Ig TBEV) and control—against Sindbis virus (Sindbis virus (SINV), genus Alphavirus), diluted in 0.05 M carbonate-bicarbonate buffer with a pH 9.6, in a volume of 100 μl per well. Sucrose-acetone antigen (SAA) served as a positive control for TBEV. In contrast, SAA derived from the brain of uninfected mice (SAA N) and SAA derived from the brain of YF infected mice served as negative controls. According to the ELISA results, the chimeric YFV 17DD-UN/TBEV virus produces TBEV antigen in studied sample (gray table cells) and did not have cross reactivity with maternal vector YFV antigen or SINV sera.

**Table 1:** Immunological characterization of the chimera YFV 17DD-UN /TBEV.

	Ig TBEV	Ig SINV
YFV 17DD-UN/TBEV	0,609^*^	0,147
SАА TBEV 1:1000	1,842	0,135
SАА TBEV 1:10000	0,568	0,123
SАА YFV 1:1000	0,280	0,142
Negative control (SАА N)	0,236	0,150

^*^Optical density with immunoglobulins

### Characteristics of the YFV 17DD-UN /TBEV infectious clone

To assess the biological properties of the chimeric virus, we studied the replication kinetics of YFV 17DD-UN/TBEV and parental strain YFV 17DD-UN. The replication kinetics of chimeric virus was evaluated after five passages in Vero E6. For this purpose, we used five various cell cultures: Vero E6, BHK-21, MA-104, Spev and LLC-MK2. The experiment lasted for 4 days, with daily sampling to determine the titer of the virus (PFU/ml) and viral load by qPCR (copies/mL) (Fig 2B). The permissiveness of all cell lines was similar for parental strain and chimeric virus. Pronounced CPE was observed during their replication. The exception was MA-104 cell line, which was less susceptible to viruses, as infection and viral replication did not result in CPE. However, for other cell lines, virus titers for YFV 17DD-UN/TBEV and YFV 17DD-UN exceeded 107 PFU/ml at 48 hours post-infection. The peak of replication rate observed on the second day (48 h) of infection in all cell lines. The level of chimeric virus YFV 17DD-UN/TBEV replication rate was lower than parental YFV 17DD-UN.

In PFU assay for YFV 17DD-UN/TBEV and YFV 17DD-UN on Vero E6 cells were observed the formation of small plaques. The average plaque diameter did not exceed 1.5 mm (Fig 2C). In general, the obtained data suggests that the phenotypic properties of the generated chimeric virus are similar to backbone vector construct YFV 17DD-UN strain.

### Experimental immunization and infection model

To assess the pathogenicity and residual neuroinvasiveness of the chimeric virus YFV 17DD-UN/TBEV, groups of BALB/c mice (each group, n = 10) were subcutaneously inoculated with the virus at doses of 10^7^, 10^6^ and 10^5^ PFU per mouse. Animals immunized with the TBE vaccine EnceVir (JSC NPO Mikrogen, Russia) and the growth medium Opti-MEM were used as control groups (each group, n = 10). Two groups of BALB/c mice (each group, n = 10) were subcutaneously inoculated with the YFV 17DD-UN and TBEV at doses of 10^7^ PFU and 10^5^ PFU per mouse respectively. We observed neurological symptoms such as paresis in TBEV group, all mice in the group had the same neurological symptoms and were sacrificed 11 days after infection. Similar symptoms were observed in group of chimeric virus, where animal received 10^7^ PFU. Unlike the TBEV group, in this group the neurological signs in individual mice in the group appeared earlier (1 mouse had paresis on day 7 after inoculation YFV 17DD-UN/TBEV 10^7^ PFU/mouse) and lasted longer (the last mouse with paresis was sacrificed on day 13 after inoculation YFV 17DD-UN/TBEV 10^7^ PFU/mouse). It should be noted that the largest number of animals with paresis was also observed on day 11 (5 mice). Brain and liver tissues were excised from the all animals sacrificed before the end of the experiment. Then tissues were examined by the qRT-PCR, as a result, YFV 17DD-UN/TBEV viral RNA (10^8^−10^9^ copies/mL) was detected in brain tissues, but not in liver.

**Fig 3:**
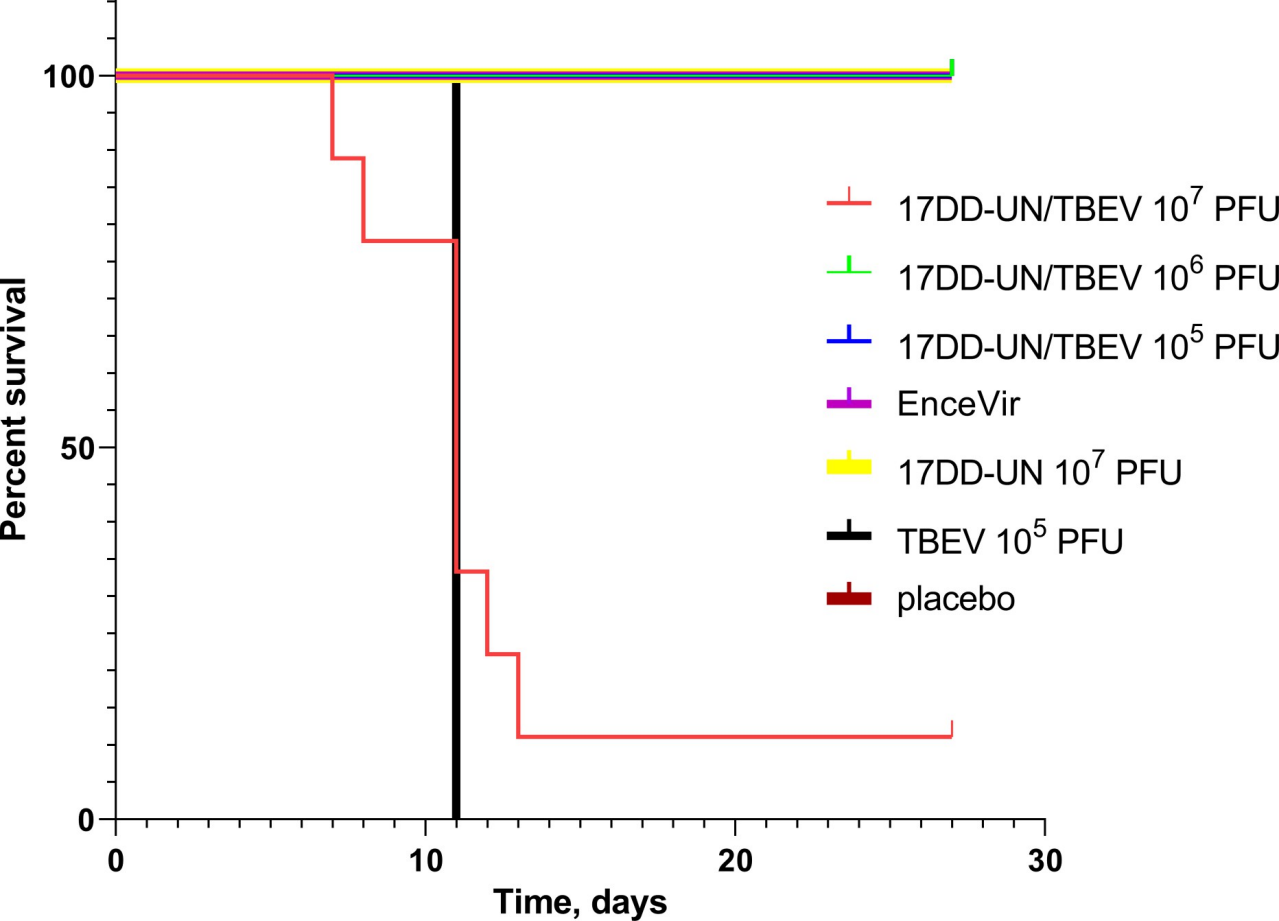
Assessment the pathogenicity and residual neuroinvasiveness of the chimeric virus YFV 17DD-UN/TBEV. Mice received YFV 17DD-UN/TBEV, EnceVir, YFV 17DD-UN, TBEV or Placebo (each group, n = 10) at the indicated doses. The mortality of animals was observed for 28 days. The statistical significance of differences among the survival curves between groups was determined using the log-rank test in GraphPad Prism 8.0.1.

However, no deterioration or neurological signs were detected in the 10^6^ and 10^5^ YFV 17DD-UN/TBEV PFU animal groups. We observed no animal deaths in the placebo group and in the vector YFV 17DD-UN, 17DD-UN/TBEV 10^6^ PFU and 17DD-UN/TBEV 10^5^ PFU groups. To interpret Kaplan-Meier survival analysis was used the log-rank (Mantel-Cox) test, which showed significant differences among the survival curves (p < 0.0001). The survival rate of animals in the 10^7^ PFU group was 11% and 100% in the 10^6^, 10^5^ PFU, EnceVir, YFV 17DD-UN and negative control groups. In the TBEV group, the survival rate was 0% (Fig 3).

**Fig 4:**
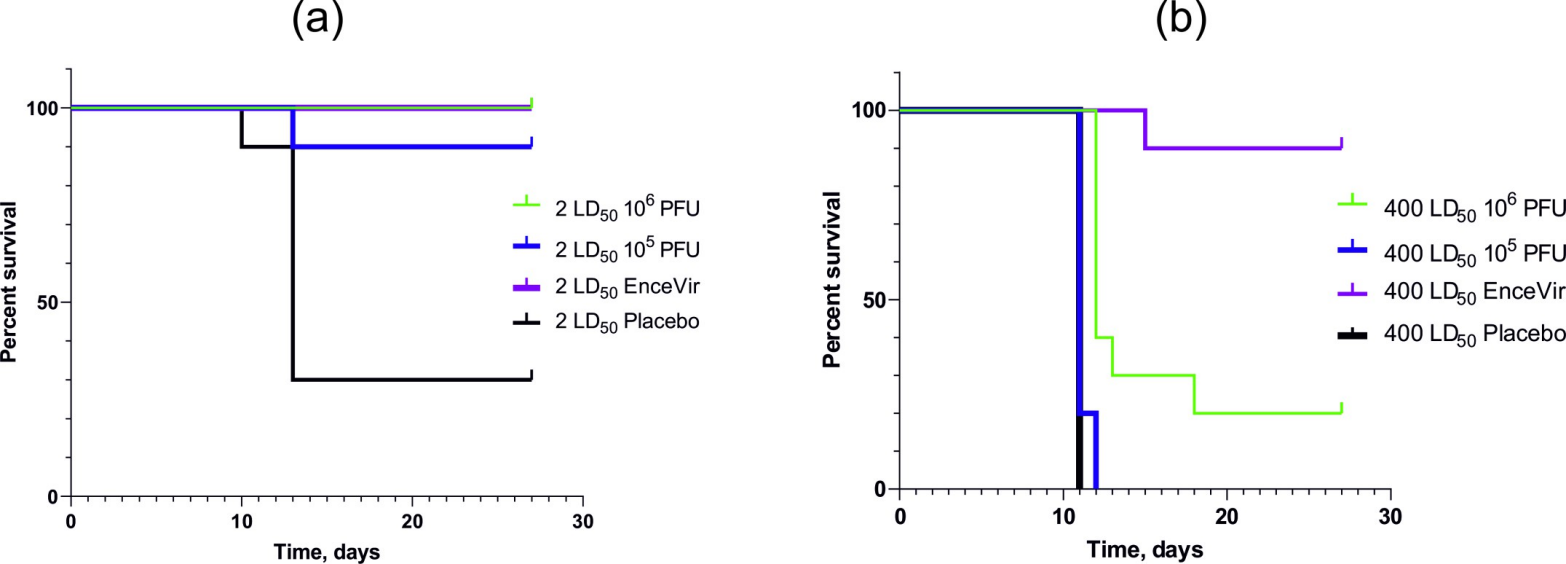
Assessment protectivity of the chimeric virus YFV 17DD-UN/TBEV. Mice received YFV 17DD-UN/TBEV, EnceVir or Placebo at the indicated doses (each group, n = 10). The mortality of animals was observed for 28 days. (a) Kaplan-Meier survival analysis immunised mice, TBEV Absettarov strain challenge by 2 LD_50_. (b) Kaplan-Meier survival analysis immunised mice, TBEV Absettarov strain challenge by 400 LD_50_.

To assess the ability of the chimeric YFV 17DD-UN/TBEV virus to protect animals from TBE, we infected the surviving animals with TBEV (strain Absettarov). Experimental infection of animals with a dose of 2 LD_50_ correlated with the 100% and 90% survival rate in the 10^6^ and 10^5^ PFU groups, respectively. The survival rate in the 10^6^ and 10^5^ PFU groups was 20% and 0%, respectively, when infected with a dose of 400 LD_50_. Significant differences among the survival curves (p < 0.0001) were showed by log-rank (Mantel-Cox) test as for 2 LD_50_ as for 400 LD_50_ (Fig 4).

To assess the neutralizing activity of serum after the immunization with the chimeric YFV 17D DUN/TBEV virus, mice (n = 6) of the BALB/c line were immunized subcutaneously with different doses of the chimeric virus. After 15 days, all animals were euthanized, and we took blood samples for the investigation. It was found that immunization of mice with a dose of 10^3^ PFU did not lead to the formation of neutralizing antibodies. The antibody titer was determined as the serum dilution that inhibited 90% of viral infection (NT_90_). NT_90_ values were based on a visual assessment on the inhibition of TBEV cytopathic effect by the sera of immunized animals. The most pronounced neutralizing activity against TBEV european subtype was found in mice immunized with a dose of 10^5^ PFU (Table 2).

**Table 2:** Serum neutralizing activity against TBEV strain Absettarov of the European subtype.

	NT_90_					
Group (PFU/mouse)	1	2	3	4	5	6
17DD-UN/TBEV (10^3^)	nd*	nd	nd	nd	nd	nd
17DD-UN/TBEV (10^4^)	1/20	nd	nd	1/10	nd	nd
17DD-UN/TBEV (10^5^)	nd	1/40	1/10	1/10	1/10	nd

*nd–not detected

## Discussion

The aim of our work was to evaluate the ease and reliability of generating vaccine candidates based on the yellow fever vector genetic backbone. Chimeric flaviviruses constructed using an attenuated strain as a base have been well studied in the development of DENV, WNV, JEV and TBEV vaccines [28–31]. In our study, we simply obtained an infectious clone of the yellow fever vector YFV 17DD-UN and hybrid YFV 17DD-UN/TBEV using the previously described ISA protocol. The resulting viruses showed high genetic and phenotypic stability during sequential passaging (5 passages) in cell culture. The phenotypic properties of the chimeric virus, such as plaque formation and replication on various cell lines, did not differ between YFV 17DD and YFV 17DD-UN/TBEV. Replication of the chimeric YFV 17DD-UN/TBEV virus decreased, reaching the plateau on the third day of infection (72 h) on almost all the studied cells. In general, the collected data suggests the preservation of the phenotypic properties of maternal vector YFV 17DD by the chimeric virus.

Developing an effective and safe live attenuated vaccine requires a precise balance between virus attenuation and immunogenicity. One of the TBEV’s displeasing properties is its neurotrophy, which makes retrieving a live attenuated viral vaccine against TBEV quite an ambitious task. It has been already shown that chimeric flaviviruses were characterized by decreasing neurotrophy properties in relation to the original virus [30–32]. In addition, specific mutations introduced into the neurovirulence loci determinants of the flavivirus genetic backbone may contribute to the attenuation of neurotrophy in mice [29, 33, 34]. As it has been heretofore shown, chimeric candidate vaccines against TBEV, including Langat/DENV-4, TBEV/DENV-4 and TBEV/JEV (ChinTBEV) had lower neurovirulence in mice and non-human primates, compared with the original viruses [30, 35, 36]. Existing neuroinvasiveness of the generated chimeric YFV 17DD-UN/TBEV virus is consistent with the data presented in the studies of other scientific groups [30, 31, 36, 37]. The neuroinvasion is most likely owing to the critical determinants in the E protein of the initial TBEV. Neurotropism is a common non-backbone related property of recombinant viruses carrying a TBEV insert. Thus, using YFV 17DD-UN as a backbone for recombinants generation makes it possible to study the residual neurotropism, neurovirulence and neuroinvasiveness of various genetic variants of the TBEV.

The successful use of hybrid flaviviruses requires the selection of the optimal antigenic composition, which allows obtaining the widest possible protective properties. In the case of TBE, it is desirable to obtain a universal antigen capable of protecting against all variants common in Northern Eurasia. At the same time, it is desirable to avoid the risks of antibody dependent enhancement (ADE) [38]. The chimeric virus developed by us can also be used as a tool for selecting the most optimal E protein, which is the main immunogen. Future experiments with reverse genetics might allow to choose universal antigen for universal TBEV vaccine development and provide data for mutating amino acid residues in order to achieve a decreased neurovirulence in mice.

In the study of the YFV 17DD-UN/TBEV immunogenicity, a dose-dependent induction of the neutralizing antibodies formation was noted as early as 2 weeks after a single immunization of mice. The average titer of neutralizing antibodies in the serum of mice immunized with a dose of 10^5^ PFU was 1/12, which can be considered as seroconversion but did not protect animals from high doses of virulent TBEV. We can explain the low titer of virus neutralizing antibodies compared to other candidate vaccines and existing vaccines against TBEV by the premature serum collection. In addition, a single immunization is not plentiful to form a high titer [30, 35, 36, 39]. Experiments with control infection showed that immunization with a high dose (10^6^ PFU) of the YFV 17DD-UN/TBEV provided reliable protection (100%) with subcutaneous administration of 2 LD_50_ TBEV. The survival rate of animals was only 25% when infected with a high dose of TBEV 400 LD_50_. The low level of protection from infection with high doses of TBEV in mice is due to the low titer of neutralizing antibodies gained from a single immunization, which also points out the need for further studies. The control EnceVir group had a survival of 90% with a high dose of TBEV 400 LD_50_, thus inactivated vaccine based on the Far-Eastern TBEV strain protects animals against TBEV strain of the European subtype. Since the vaccines must protect against the entire spectrum of virus variants circulating in nature, the control of the protective activity of a generated vaccine (inactivated and live attenuated) should include different TBEV strains and subtypes. The chimera generated by us has a relatively low immunogenicity. The obtained results show that the study of the structure of the chimera virions and the features of the formation of the immune response is required to understand the reasons for the low immunogenicity and protectiveness of the chimeric virus. Similar results were obtained in the study of tick- and mosquito-borne flavivirus Langat/dengue4 and TBEV/JE vaccine strain chimeras. The chimera we have obtained will be a good tool for these studies [30, 35, 39, 40].
